# Clinical and mutational features of Vietnamese children with X-linked agammaglobulinemia

**DOI:** 10.1186/1471-2431-14-129

**Published:** 2014-05-28

**Authors:** Quang Van Vu, Taizo Wada, Huong Thi Minh Le, Hai Thanh Le, Anh Thi Van Nguyen, Ohara Osamu, Akihiro Yachie, Sang Ngoc Nguyen

**Affiliations:** 1 Department of Pediatrics, Haiphong University of Medicine and Pharmacy, 72 A Nguyen Binh Khiem, Ngo Quyen, Haiphong, Vietnam; 2 National Hospital of Pediatrics, Hanoi, Vietnam; 3 Department of Pediatrics, Intistute of Medical, Pharmaceutical and Health Science, Kanazawa University, Kanazawa, Japan; 4 Kazusa DNA Research institute, Chiba, Japan

**Keywords:** X-linked agammaglobulinemia, XLA, Bruton tyrosine kinase (*BTK*), Hypogammaglobulinemia, Mutation analysis, Bruton disease

## Abstract

**Background:**

X-linked agammaglobulinemia (XLA) is a primary immune deficiency characterized by recurrent bacterial infections and profoundly depressed serum immunoglobulin levels and circulating mature B cells. It is caused by mutations of the Bruton tyrosine kinase (*BTK*) gene and is the most common form of inherited antibody deficiency. To our knowledge, this is the first report of XLA from Vietnam.

**Methods:**

We investigated the *BTK* gene mutations and clinical features of four unrelated Vietnamese children.

**Results:**

The mean ages at onset and at diagnosis were 2.5 and 8 years, respectively. All patients had a medical history of otitis media, pneumonia, and septicemia at the time of diagnosis. Other infections reported included sinusitis, bronchiectasis, arthritis, skin infections, meningitis, and recurrent diarrhea. We identified one previously reported mutation (c.441G >A) and three novel mutations: two frameshifts (c.1770delG and c.1742 delG), and one nonsense (c.1249A >T).

**Conclusions:**

The delayed diagnosis may be attributable to insufficient awareness of this rare disease on the background of frequent infections even in the immunocompetent pediatric population in Vietnam. Our results further support the importance of molecular genetic testing in diagnosis of XLA.

## Background

X-linked agammaglobulinemia (XLA, OMIM 300300), first described by Bruton in 1952, is a fully penetrant X-linked recessive disorder characterized by recurrent bacterial infections, profound hypogammaglobulinemia and marked decrease in the number of B cells in the presence or absence of positive family history. It occurs in approximately one in 200 000 individuals [1-5]. The gene responsible for XLA is the Bruton Tyrosine Kinase (*BTK*) gene mapped to the long arm of chromosome X in the region of Xq 21.3- q 22 [2,4,6-8]. The *BTK* gene is expressed in B cells and moncytes throughout their differentiation but not in T cells [9]. The gene contains 19 exons and encodes a protein with five functional domains: plekstrin homology (PH) domain, Tec homology (TH) domain, Src homology 3 (SH3) domain, Src homology 2 (SH2) domain and catalytic (SH1) domain [3-5]. According to the *BTK* database (http://rapid.rcai.riken.jp/RAPID/mutation?pid_id=AGID_8), at present 592 unique *BTK* mutations have been found in XLA patients. These mutations are found in both exons and introns throughout the gene and may result in complete absence of protein, or non functional proteins [4].

There have been very few reports of XLA from developing countries [10,11]. In Vietnam, after many years in war and in low socio-economic conditions, we usually make a diagnosis of XLA based on clinical manifestations, family history, hypogammaglobulinemia, and low numbers of circulating B cells, but not genetic analysis. In this study, we report for the first time 4 Vietnamese boys with XLA, confirmed by mutation analysis of the *BTK* gene in an attempt to improve the diagnosis and management of XLA in Vietnam.

## Methods

### Patients

Patient 1 presented with sepsis and erysipelas at age of 6 years. Several episodes per year of sinusitis, which were treated by antibiotics for at least 2 months, were noted between the age of 7 and 9 years. Patient 1 was diagnosed as XLA at the age of 10 years. There was no lymphadenopathy and his tonsils were absent. Patient 1 had an elder male sibling who died at 6 years old due to recurrent pneumonia and purulent meningitis. Chest X-ray showed lobar pneumonia in the left lung. No organism could be isolated.

Patient 2 was well for the first 8 months of life. He then had frequent pneumonia and sore throats. From 1 to 5 years of age, he had 3–4 episodes of otitis media, 4–5 episodes of pneumonia, and 1–2 episodes of erysipelas every year, which were treated by antibiotics for at least 10 days. At the age of 6 years, patient 2 was referred to the National Hospital of Pediatrics because of sepsis, gastrointestinal hemorrhage, otitis media, and pneumonia. His tonsils were hypoplastic. He had an elder male sibling who was died at 9 years of age due to recurrent infections, including pneumonia, otitis media, and dermatomyositis. Patient 2 had an elder sister who was well.

From 9 months of age, patient 3 had recurrent otitis media and mastoiditis that were operated on three times (4, 7, and 9 years of age) at the National Hospital of Pediatrics. However, he did not fully recover after the operations. At 11 years old, patient 3 was referred to the National Hospital of Pediatrics again due to septicemia and purulent meningitis. His tonsils were absent. He had no male sibling in the family and his female sibling was normal.

Patient 4 was noted to have persistent diarrhea and skin infections when he was 18 months old. From 2 to 4 years of age, he had 5–6 episodes of otitis media per year. At 5 years of age, he was admitted to the National Hospital of Pediatrics because of septicemia, persistent coughing and otitis media and left knee arthritis. His tonsils could not be visualized. His mother had a history of lupus and two female siblings were normal.

Written informed consent for publication of these case reports and accompanying images were obtained from the patients’ parents. Copies of the signed informed consent forms are available for review by the Editor of BMC Pediatrics. Approval for the study was obtained from Medical Ethics Council of Haiphong University of Medicine and Pharmacy, and informed consent was obtained according to the Declaration of Helsinki.

### BTK mutation detection

We applied DNA sequencing protocols of the *BTK* gene standardized at the Department of Human Genome Research, Kazusa DNA Research Institute (http://rapid.rcai.riken.jp/RAPID). In brief, genomic DNA was isolated from whole blood by spin column purification (QIAGEN, Valencia, CA, USA). All 19 exons of the *BTK* gene and the exon-intron boundaries were amplified in individually or in group yielding amplicons. Primer sequences are available online (http://rapid.rcai.riken.jp/RAPID/dnaseq?pid_id=AGID_8).

Polymerase chain reactions (PCR) were carried out in 10 μl containing 0.5 μl of genomic DNA, 5 pmol of each primer, 5 μl of Multiplex PCR mix 2, 0.05 μl of Multiplex PCR Mix 1 (TaKaRa Multiplex PCR assay Kit™; Takara, Shiga, Japan). Cycling profiles used for all reactions consisted of one hold at 94°C for 30 seconds followed by 30 cycles at 94°C for 30 seconds, 55°C for 10 seconds, 72°C for 60 seconds, with a 5 minutes final extension at 72°C.

PCR products were purified by the ExoSAP-IT (USB, Cleveland, OH, USA) according to the manufacturer’s protocol. Sequencing was performed using the BigDye deoxyterminator v3.1 cycle sequencing kit (Applied Biosystems, Foster City, CA, USA) with an automated ABI 3130 DNA sequencer. Detected mutations were confirmed by sequencing in the opposite direction. Numbering of nucleotide and amino acid position refers to cDNA sequence (GenBank accession number NM_000061.1), where the A of ATG translation initiation start site represents nucleotide +1. Intron sequence information was obtained from reference GenBank sequence (NG_ 009616.1) [2-4].

## Results

### Delayed diagnosis and severe recurrent bacterial infections

The clinical data collected prior to diagnosis are shown in Table 1. The mean age at onset and at diagnosis was 2.5 and 8 years, respectively. All patients showed recurrent infections; otitis media, pneumonia, and septicemia were universally observed, followed by sinusitis (2/4), bronchiectasis (2/4), arthritis (2/4), skin infection (2/4), meningitis (2/4), and recurrent diarrhea (1/4). Tonsils were absent or hypoplastic in all patients. Patients 1 and 2 had a positive family history. Their brothers died of recurrent infections at age of 6 and 9 years, respectively. No patients with a positive family history were screened for XLA before onset of infection.

**Table 1:** Clinical pictures of 4 XLA patients

**Patient**	**1**	**2**	**3**	**4**
Age at onset (y)	6	1	0.8	2
Age at diagnosis* (y)	10	6	11	5
Present age (y)	13	8	15	Died at 8 Y
Family history	+	+	-	-
First symptom	Septicemia	Otitis media	Otitis media	Otitis media
Otitis media	+	+	+	+
Sinusitis	+	-	+	-
Pneumonia	+	+	+	+
Bronchiectasis	+	-	+	-
Arthritis	+	-	-	+
Skin infection	+	+	-	-
Septicemia	+	+	+	+
Meningitis/encephalitis	+	-	+	-
Recurrent diarrhea	-	-	-	+
Tonsils	Absent	Hypoplastic	Absent	Hypoplastic
Duration of IVIG treatment (m)	25	17	33	12

*y*, years; *m*, months; *IVIG*, intravenous immune globulin; +, yes; -, no.

*Age of confirmed agammaglobulinemia.

### Low levels of circulating B cells and immunoglobulins

As shown in Table 2, all patients exhibited very low serum immunoglobulin levels at diagnosis. No patients received intravenous immunoglobulin (IVIG) substitution therapy before diagnosis. The percentage of circulating B cells was 0–1.9%. Based on these clinical and laboratory findings, our patients were clinically diagnosed with XLA. Neutropenia was observed in patient 4. The ratio of CD4^+^ to CD8^+^ T cells was markedly inverted in patients 1 and 4.

**Table 2:** Immunological features of 4 XLA patients

**Patient**		**1**	**2**	**3**	**4**	**Reference values**
WBC (×10^9^/L)		15.1	9.4	8.8	4.6	5-10
Neutrophils (×10^9^/L)		11.2	5.1	4.3	2.1	1.5-5
Lymphocytes (×10^9^/L)		3.7	4,2	4.4	2.4	1.5-5
Ig (g/l)	IgG	0.06	2.18	1.7	0.01	6-15
	IgA	< 0.01	0.03	0.02	0.01	1.5-2.25
	IgM	< 0.01	0.21	0.06	0.13	0.75-1.5
CD19^+^ (%) (/μL)		< 0.1	< 0.1	< 0.1	1.9 (58)	6-25
CD3^+^ (%) (/μL)		95 (3535)	93 (3564)	89 (3982)	95 (2335)	55-84
CD4^+^ (%) (/μL)		29 (1078)	45 (1707)	36 (1616)	15 (367)	31-60
CD8^+^ (%) (/μL)		59 (2209)	37 (1404)	47 (2102)	73 (1792)	13-41
CD4^+^/CD8^+^		0.49	1.22	0.77	0.2	0.9-3.1

*WBC*, white blood cells; *Ig*, immunoglobulin.

### Three novel and one reported mutations of the *BTK* gene

To confirm the diagnosis, mutation analysis of the *BTK* gene was performed (Table 3). Patients 1 and 3 were found to carry novel frameshift mutations, c.1770delG and c.1742delG, respectively. They were located in the SH1 domain. Patient 2 had a novel nonsense mutation (c.1249A > T), which was located in the SH2 domain. The prevalent silent polymorphism affecting (c.1899 C > T) was also observed in patient 2. Patient 4 carried a reported mutation (c.441G > A) in the TH domain.

**Table 3:** BTK mutation analysis in 4 XLA patients

**Patient**	**Exon/Intron**	**Mutation**	**Protein domain**	**Protein alteration**	**Mother status**
1	Exon 18	c.1770delG	SH1	p.Gly594fsX54	N.D.
2	Exon 14	c.1249A > T*	SH2	p.Lys417X	N.D.
	Exon 18	c.1899C > T**	SH1		
3	Exon 17	c.1742delG*	SH1	p.Ala582LeufsX4	Carrier
4	Exon 6	c.441G > A	TH	p.Trp147X	N.D.

*Novel mutation; **silent polymorphism; *N.D*., not done; *SH1*, catalytic kinase domains; *SH2*, Src homology 2 domain; *TH*, Tec homology domain.

## Discussion

XLA is caused by a B-cell developmental defect. Being an arrest of differentiation of pre-B cells to mature B cells, pre-B cells are found in normal amounts while mature B cells are nearly undetectable, that result in a pronounced reduction of serum Ig of all classes. Therefore, affected boys suffer from recurrent bacterial and enteroviral infections after six months of life after maternal antibodies are no longer present in the infant’s circulation. Mutations in all five domains of the *BTK* gene have been found to cause XLA [6,12-16]. In the present study, we report four Vietnamese boys with XLA and investigated the mutation characteristics of their *BTK* gene.

The clinical manifestations of our XLA patients were typical and similar to previous reports [5,8,10,11], with severe recurrent infections (Table 1), low levels of mature B cells and serum immunoglobulin (Ig) (Table 2). Pneumonia, septicemia, otitis media were observed in all our patients. Bronchiectasis was present in patients 1 and 2, possibly reflecting the delay of diagnosis with a complication of acute pneumonia in their histories. Patients 1 and 4 presented with arthritis, which was treated as juvenile idiopathic arthritis before diagnosis of XLA; both patients had markedly inverted CD4^+^/CD8^+^ ratio (Table 1). However, a relationship between inverted CD4^+^/CD8^+^ ratio and arthritis is presently unclear. Similar to previous reports [8,10,11], all our patients were diagnosed with XLA late. The age of onset of infection was between 8 months and 6 years (mean 2.5 years). Nonetheless, the age of diagnosis of XLA ranged from 5 to 11 years old (mean 8 years), possibly reflecting the poor knowledge of health workers about XLA and insufficient equipment for diagnosis of XLA in Vietnam, such as flow cytometer. Moreover, the delayed diagnosis may be because the disease is rare and infections (such as pneumonia, sinusitis, and otitis media) are common in our pediatric population. As the estimated prevalence of XLA is 1/200 000 [8], it is suggested that many XLA cases in Vietnam may be undiagnosed. Because early initiation of immunoglobulin replacement therapy could prevent sequelae associated with infections, early diagnosis is of great importance. National networks and diagnostic guidelines for XLA may be helpful for us to improve these issues.

Our XLA patients began to be treated with prophylactic IVIG and appropriate antibiotics for acute and chronic infections. Patients 1 and 2 were free from serious infections with 400 and 300 mg/kg of IVIG replacement therapy every 4 weeks, respectively. Patient 3 developed serious infections frequently while he was receiving 300 mg/kg of IVIG every month but was well after the dose had been increased to 450 mg/kg. Patient 4 was well after 5 months on 400 mg/kg of IVIG therapy every month; however, his family decided to use traditional medicines instead of IVIG at age of six. Patient 4 was hospitalized again with multiple complications of serious infections; he died after 7 months despite reintroduction of treatment with IVIG and antibiotics. Our results suggest that IVIG replacement represents an important role to improve the prognosis of XLA [10,11,17].

Our patients were examined for the presence of *BTK* gene mutations. Their mutations were distributed in both coding and non-coding (data not shown) regions of the *BTK* gene, which were compatible with the range of mutation as previously described [3-5,18,19]. Three out of 5 *BTK* mutations (60%) were located on SH1 domain (Table 3). In accordance with previous reports [4,5,8,20], their *BTK* mutations affected mainly SH1 domain; this may be explained by the length of SH1 domain. There was no correlation between clinical phenotype and the mutation’s site, suggesting that other factors are contributing to the phenotype and affecting the severity of disease, such as other components of the *BTK* mediated signaling pathway (μ heavy chain, λ5) and variants in proteins that function as part of the innate immune system [1,2,21]. Environmental and potentially epigenetic factors may also contribute to the phenotype. We have analyzed the XLA carrier status in mother of patient 3 and found the same mutation. Because of the variability in clinical phenotype, the diagnosis of XLA may be difficult in some cases with atypical phenotype or without family history of disease. In such cases, molecular genetic testing provides an important tool for XLA confirmation and may allow accurate carrier detection [2,5,8].

One out of 4 patients (25%) developed neutropenia during the course of the disease. Several previous studies have reported that about 10-27% of XLA patients might be associated with neutropenia including mild, moderate and severe neutropenia [22-25]. Patient 4 had two episodes of moderate neutropenia before and at the time of diagnosis of XLA (Table 2). The neutropenia durations of patient 4 were less than one week and resolved completely after two weeks of treatment with antibiotic and IVIG therapy suggesting the role of infections inducing neutropenia [22,26]. This phenomenon can most probably be explained by reactive oxygen species- mediated apotosis of neutrophils triggered by the engagement of innate receptors and not by abnormal myeloid differentiation [27].

## Conclusions

We describe four Vietnamese boys with typical phenotype of XLA but delayed diagnosis. Our patients were definitively diagnosed by genetic analysis. Their mutations are three novel and one recurrent *BTK* mutations. This is the first Vietnamese study describing clinical features and *BTK* mutations in patients with XLA. The delayed diagnosis may be attributable to insufficient awareness of this rare disease on the background of frequent infections even in the immunocompetent pediatric population in Vietnam.

## Abbreviations

XLA: X-linked agammaglobulinemia; BTK: Bruton tyrosine kinase; TH: Tec homology domains; SH2: Src homology 2 domains, SH1, catalytic kinase domains; IVIG: Intravenous immunoglobulin.

## Competing interests

The authors declare that they have no competing interests.

## Authors’ contributions

QVV, SNN participated in study design, protocol development and performance, data analysis, interpretation of data and writing of the manuscript. HTML, HTL, ATVN, and OO carried out the clinical data collection and data analysis. TW, AY reviewed and revised the manuscript making important intellectual contributions. All authors read and approved the final manuscript.

## Pre-publication history

The pre-publication history for this paper can be accessed here:

http://www.biomedcentral.com/1471-2431/14/129/prepub
